# On the financial viability of negative emissions

**DOI:** 10.1038/s41467-019-09782-x

**Published:** 2019-04-16

**Authors:** Johannes Bednar, Michael Obersteiner, Fabian Wagner

**Affiliations:** International Institute for Applied Systems Analysis, Laxenburg, A-2361 Austria

## Abstract

Climate mitigation will have significant impacts on government spending necessary to finance large-scale deployment of Negative Emission Technologies (NETs). The required expenditure might consume up to a third of general government expenditure in advanced economies.

## Abstract (web summary)

Recent publications have raised concerns regarding the actual feasibility Negative Emission Technologies (NETs). Here the authors commented on the financial viability of large-scale late century NETs and suggested that expenditure peak will occur in the end of the century, which would require massive global subsidy program.

The Paris Agreement aims to limit global temperature increase to 2 °C above preindustrial levels and to balance GHG sources and sinks in the second half of this century. The technical feasibility of these targets has broadly been demonstrated by the 5th Assessment Report (AR5) of the Intergovernmental Panel on Climate Change (IPCC). Recent publications, however, raise concerns about the broader political and economic feasibility of compatible emission trajectories, which typically rely on large-scale deployment of Negative Emission Technologies (NETs)—a type of pilot backstop technology that is often associated with enormous amounts of natural land loss, stranded assets by 2100, a potentially dangerous emission overshoot level and resulting fundamental ethical issues of intergenerational equity^1–4^.

Here, we argue that the financial viability of late-century NETs has thus far not been adequately addressed and show that NETs enter IPCC scenarios for the wrong (discounting), not for the right reason (hedging uncertainties).

## NETs will require public subsidies

Integrated Assessment Models (IAMs) foresee large-scale late-century NETs in most AR5 2 °C scenarios^5^, while being silent about potential sources of funding for an atmospheric GHG restoration Manhattan Project. In 2060 CO_2_ emissions will have declined to 30% of present levels and BECCS will have scaled up to 50% of maximum deployment (see SI D). These numbers are 13% and 85%, respectively, for the schematic intensive economy pathway of the IPCC’s Special Report, making the fossil sector an inadequate source of funding in line with a contemporary Polluter Pays Principle, e.g., through ear-marked tax recycling to negative emissions, already by 2060. By then cumulative emissions will have overshot the carbon budget, so that NETs remain the only option for returning to the Paris targets. In the absence of private incentives for atmospheric carbon removal, large-scale deployment of NETs will have to be publicly subsidized. A first-order estimate of the scale of government expenditures can be derived from the carbon price and net CO_2_ emissions reported for most AR5 scenarios: volume times price gives revenues, however, a tax on negative emissions turns into a government expenditure item.

**Fig. 1:**
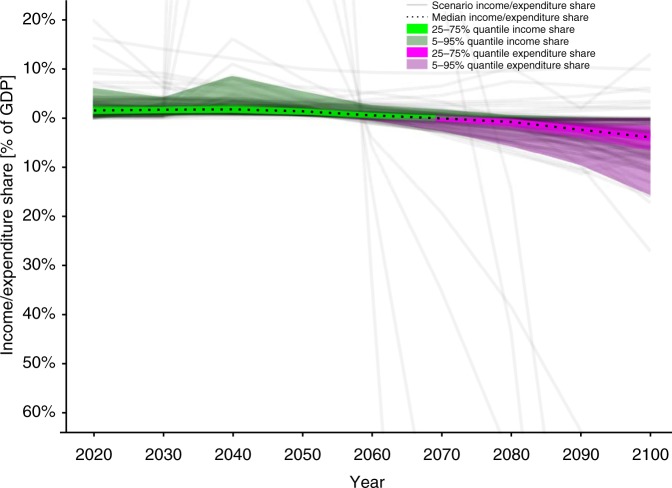
Public income and expenditure as GDP-percentage generated by a carbon tax on net CO_2_ emissions. Income/expenditure shares are derived from net CO_2_ emissions, carbon price and GDP reported by AR5 scenarios compatible with the Paris agreement temperature target of 2 °C (see SI A). Positive net emissions result in tax income which gradually turns into a subsidy as net CO_2_ emissions become negative with increasing deployment of Negative Emission Technologies. The uncertainty range  mainly results from different carbon price levels as consequence of diverging model characteristics and scenario setups (e.g., choice of technology mix, carbon budget and socio-economic factors)

**Fig. 2:**
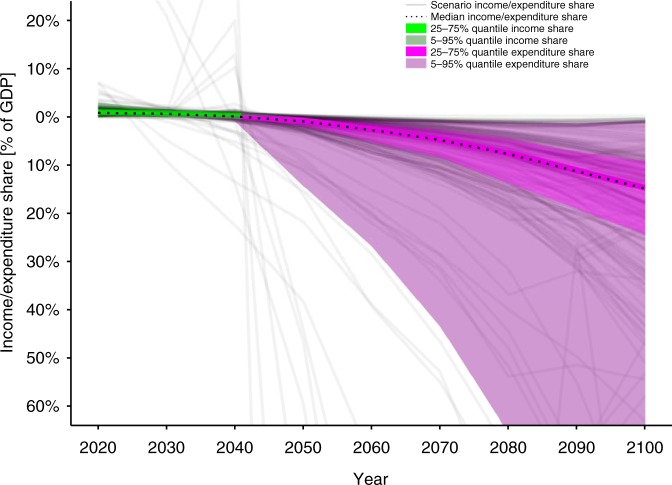
Income and expenditure shares for ANNEX I countries (UNFCCC) under a differentiated burden sharing arrangement. Income/expenditure shares as GDP-percentage generated by a carbon tax on net CO_2_ emissions are derived from net CO_2_ emissions, carbon price and GDP reported by AR5 scenarios compatible with the Paris agreement temperature target of 2 ^o^C and redistributed taking into account historical carbon emissions (see SI A and B for details). Tax income turns into a subsidy before mid-century which peaks at 15% of GDP in 2100. The uncertainty range mainly results from different carbon price levels as consequence of diverging model characteristics and scenario setups (e.g., choice of technology mix, carbon budget and socio-economic factors)

Carbon prices increasing at rates above economic growth lead to small near-term revenues compared to future expenditures for NETs – even when expressed as shares of GDP – as demonstrated in Fig. 1 . At zero transaction costs of a uniform globally applicable carbon pricing mechanism, median income reaches a maximum of 1.8% of global GDP in 2040. In 2070, it turns into a subsidy peaking in 2100 at 3.9% of GDP—higher than the US’ current expenditure share for defense. Similar trends hold for the RCP2.6 SSP^6^ and 400–1000 GtCO_2_ CD-LINKS^7^ scenarios, peaking at 1.6% and 4.1% of GDP in 2100, respectively. Cost allocation following considerations of intra- and intergenerational effort sharing in line with the Brazilian Proposal^8^ would lead to public spending peaking at 15% of GDP in Annex I countries (UNFCCC) as depicted in Fig. 2, rendering the implementation of this mechanism extremely difficult (see SI B). The US’ incomplete participation in global mitigation efforts (see SI C) could result in a subsidy of 13% by 2100 for the rest of the world. However, such scenarios would likely be characterized by alternative sets of optimal solutions with even higher mitigation costs.

## Expenditure peaks in 2100

Discounting at 4–5% over 80–90-years leads to substantial Net Present Cost (NPC) reductions if mitigation (emission reductions and negative emissions) can be deferred—which is significantly amplified by NETs. Conversely, in scenarios that limit NETs mitigation costs are incurred much earlier and are therefore larger in present terms. For instance, limited supply of bioenergy (BE) or Carbon Capture and Storage (CCS) leads to net present mitigation costs 64% and 138% above corresponding levels in scenarios with unlimited amount of BECCS^9^. In contrast, limited solar/wind or nuclear energy results in only 6–7% cost increase. In IAMs, mitigation costs are reflected in carbon prices driven by the level of ambition (1.5 ^o^C versus 2 ^o^C), or by technological or socio-economic challenges hindering mitigation. They emerge from explicit endogenous or exogenous carbon pricing instruments, or from shadow prices of emission budget constraints. In the latter case they reflect the marginal increase of the objective function when tightening the carbon budget for an infinitesimal unit. Since costs are discounted in the objective function, the shadow price increases with the discount rate to obtain the carbon price over time. Due to discounting, NETs and carbon prices typically peak in 2100 resulting in a subsidy borne by a global society much richer than today. However, since economic growth is projected below the discount rate, expenditure shares are bound to increase even under constant levels of negative emissions.

## Discussion

A carbon tax is one possible instrument for the achievement of emission constraints in models which are silent about alternative economic and political mechanisms. Our legitimate interpretation of the carbon price as subsidy is, however, different from the costs of NET projects that generate a rent under inflated price levels resulting from deployment obstacles (e.g., limited deployment growth rates or absolute caps). In perfect foresight/intertemporal optimization such rents are only positive over the whole planning horizon. Conversely, myopic/recursive dynamic models are characterized by a separate optimization (and shadow price) in each time step, possibly offering a more accurate reflection of marginal costs of emission reductions. Prices are therefore to some degree artificial and depend on the specific model implementation. However, a real NET subsidy will also have to consider rents to attract the required investments, even though this might be seen as loss to society.

We considered subsidies for negative emissions (provided by the energy and land use sectors) partly being compensated by other sectors (especially industry and transport). Full tax recycling will, however, be limited by distributional considerations. Additional contributions from taxation of non-CO_2_ GHG-emissions resulting from our agricultural practices, demography and dietary patterns^10^ were not considered, given welfare aspects of increased agricultural commodity prices notably in developing regions^11^ and the size of the agricultural sector, currently generating value added of about 3.5% of global GDP^12^.

Cost optimizing IAMs have not succeeded in generating robust mitigation strategies addressing technological and climatic uncertainties. Instead of leading to careful improvement of existing strategies, the implementation of additional, independently scalable technologies (e.g., Solar Radiation Management or Direct Air Capture) can lead to significant NPC reductions and cause radical shifts of mitigation action further towards 2100. In contrast to the initial purpose of NETs to manage climate risk^13^, as well-timed cost-saving options they tend to increase risk considering the growing evidence of negative impacts potentially following an emission overshoot. Detrimental climate feedbacks into the economy can, moreover, lead to higher relative subsidy levels required for their own, massive deployment.

## Conclusion and reflections

We arrive at three conclusions which can serve as starting points for discussing the future role of NETs in model simulations as well as in the political sphere: First and foremost, the current treatment as convenient cost-optimizing measures does not take advantage of NETs over merely zero-emission mitigation technologies, namely the potential to manage an uncertain climate. NETs need to be recognized as tool for hedging against uncertainties originating from our understanding of carbon cycle climate interactions, the participation of actors in future climate agreements, or the effectiveness of mitigation policies. For instance, the carbon budgets used in AR5 simulations were approximated by a linear function from climate model calculations and ignore many of the non-linear feedbacks of the earth system, such as permafrost thawing^14^. Treating these budgets as uncertain^13^ provides one raison d’être for NETs. Changing their role probably implies earlier and more radical mitigation than current IAM simulations suggest^15^, including near-term development and ramping-up of NETs to clarify the actual potential and scaling properties of specific pilot technology-options and to avoid their non-realization, as well as social costs and financial risks associated with late and massive CO_2_ removal.

Second, the scale of public subsidies associated with the implementation of the dominant IPCC mitigation strategy by means of a carbon price needs to be better understood, including the identification of compensatory sources of funding to minimize the burden on government budgets. Significant funds could be collected in the near-term from carbon emitters, however, at the cost of limiting means to address considerations of distribution or competitiveness. Hence, alternative market and non-market-based mechanisms for incentivizing NET deployment at the required scale will have to be developed, focusing on the refinement of tools enabling non-contemporary transfers from present polluters to future negative emissions.

Third, discounting has a distinct timing effect on deployment strategies in cost-minimizing IAMs as it leads to a delay of mitigation to the last possible moment. Unlike in dynamic welfare optimizing IAMs that take into account intermediate climate responses (and typically use lower discount rates), negative impacts of an overshoot are not part of the objective function, resulting in a strong reliance on negative emissions. We observe an inconsistency within AR5, which dedicates a whole chapter to the link between discounting and ethical concepts, e.g., via the Ramsey equation, but at the same time accepts a static discount rate across highly diverse sets of scenarios. Given the overall objective of identifying pathways of expedient action towards the Paris target, the IAM modeling communities will need to reconsider how discounting is being used in models that are rich in technologies, poor in social objectives, and yet are used to describe very different social realities, laid out, e.g., in the shared socio-economic pathways.

## Supplementary information

Supplementary Information
