# Supplementary material for: On the financial viability of negative emissions
Source: Nat Commun. 2019 Apr 16;10:1783. doi: 10.1038/s41467-019-09782-x (PMC6467865; doi:10.1038/s41467-019-09782-x)
Supplement: Supplementary file 1 — Supplementary Information [file 41467_2019_9782_MOESM1_ESM.docx]

**Technical note on the numerical calculations of expenditure shares**

1. **Data**

The scenarios for our analysis were downloaded from the AR5, SSP and CD-LINKS databases (publicly available at <https://tntcat.iiasa.ac.at/AR5DB/>, <https://tntcat.iiasa.ac.at/SspDb/>.and <https://db1.ene.iiasa.ac.at/CDLINKSDB/>) From the AR5 database we incorporated all scenarios that achieve the 2 degree Paris temperature target in 2100 (global mean surface temperature median value calculated using MAGIC6) or a total radiative forcing of 2.6 W/m^2^ where temperatures are not available. Scenarios without carbon prices and/or GDP were discarded, resulting in 186 scenarios from the Integrated Assessment Models GCAM 2.0, 3.0 and 3.1, IMAGE 2.4, MERGE-ETL_2011, MERGE_EMF27, MESSAGE V.3 and V.4, as well as REMIND 1.4 and 1.5. From the SSP database we used all 18 RCP2.6 scenarios from the models AIM/CGE, GCAM4, IMAGE, MESSAGE-GLOBIOM, REMIND-MAGPIE and WITCH-GLOBIOM. From the CD-LINKS database we incorporated 12 scenarios based on carbon budgets of 400 Gt CO_2_ (>66% chance for staying below 1.5^o^C) and 1000 Gt CO_2_ (>66% chance for staying below 2^o^C) from the models AIM/CGE 2.1, IMAGE 3.0.1, MESSAGEix-GLOBIOM 1.0, POLES CD-LINKS, REMIND-MAgPIE 1.7-3.0 and WITCH-GLOBIOM 4.4.

GDP expenditure and income shares are calculated for each set of scenarios by multiplying carbon price and net CO2 emissions and by dividing by GDP. Global GDP is reported on market exchange rate (MER) basis.

1. **Historical burden sharing**

Determining contributions to global mitigation efforts based on historical responsibilities for climate change is a far reaching and controversial topic that we address in a strongly simplified manner. The choice of an appropriate indicator to measure these contributions is a key issue and marks a trade-off between the indicator’s potential to act as a proxy for climate damages and its degree of certainty. While climate response indicators (e.g. global average surface temperature), are closely related to climate damages, estimation of such indicators is associated with a higher degree of uncertainty than, for instance, past cumulative emissions. The suggestion of the Brazilian delegation for allocating responsibilities to achieve emission reductions among countries listed in the ANNEX I of the UN Framework Convention on Climate Change (OECD countries and economies in transition) is based on individual countries’ contributions to average surface temperature calculated from cumulative historical emissions of Kyoto greenhouse gases (GHGs) starting in 1840. In addition to determining an adequate indicator there are technical and ethical considerations, for example to what extent emissions that occurred before climate change became a well-established phenomenon in scientific and political spheres should be accounted for; if emissions should be allocated production or consumption based; what emission sources and GHGs to include; or to what extent population numbers or poverty measures should be considered. All these factors can have significant implications for historical responsibilities of individual countries.

For demonstration purposes a simplified methodology as proposed by Mueller et al^1^ is justifiable. Responsibility shares for climate damages are directly assigned in accordance with past Kyoto GHG emissions and without computing potentially non-linear climate responses (such as temperature change). We construct new net-CO_2_ emission curves for each AR5 scenario reflecting the increased effort borne by ANNEX I countries, as well as the reduced efforts done by the rest of the world (ROW). The new emission pathways are constructed as follows:

Responsibility for future climate damages equals the respective group of countries’ share of past cumulative Kyoto GHG emissions since 1890, which is roughly 55% for ANNEX I countries^1^. The mitigation effort required by a group of countries is the global mitigation effort times the responsibility share (*r*). The global mitigation effort (*M_global_*) is defined as the emission reductions required from baseline scenario values (*CO2_base_*) to achieve the net-emission trajectory of the respective Paris compatible scenario (*CO2_scen_*):

$$r=0.55$$

$$M_{global}=\sum_{t=2020}^{2100} \left( {CO2}_{base}\left( t \right)- {CO2}_{scen} \left( t \right) \right)$$

$$M_{ANNEXI}=r* M_{global}$$

The share of future global baseline emissions of a group of countries equals their current emission share (*ces*), which is approximately 37% for ANNEX I countries in 2014^2^ (fossil fuel only). This share of baseline emissions is the basis for calculating emission reductions. For the rest of the world (ROW) with lower future mitigation efforts this trajectory is constructed by linear combination of the remainder (63%) of baseline and scenario emissions, which should be close to each other in the near term, and divert substantially in the future:

$$ces=0.37$$

$${CO2}_{ROW}\left( t \right)=\left( 1-ces \right)*\left( a*{CO2}_{base}(t)+\left( 1-a \right)*{CO2}_{scen}(t) \right)$$

$$s.t.:$$

$${\left( 1-r \right)* M}_{global}=\sum_{t=2020}^{2100} \left( {\left( 1-ces \right)* CO2}_{base}\left( t \right)- {CO2}_{ROW} \left( t \right) \right)$$

$$0<a<1$$

Resulting in:

$$a= \frac{r-ces}{1-ces}$$

ANNEXI CO_2_ emissions are calculated as follows:

$${CO2}_{ANNEXI}\left( t \right)={{CO2}_{scen}\left( t \right)- CO2}_{ROW} \left( t \right)$$

On a global scale, emissions as well as expenditure and income generated from the carbon pricing mechanism are unchanged if *CO2_ROW_* and *CO2_ANNEXI_* are summed up. Figure 1 depicts global baseline emissions (*CO2_base_*, blue), emission shares of ANNEX I countries (*CO2_ANNEXI_*, red) and ROW (*CO2_ROW_*, green), as well as the sum of the two, global Paris compatible emissions (*CO2_scen_*, black). ANNEX I countries start from a lower level and become net-negative in around 2040, while the effort of ROW is to hold emissions more-or-less constant at 2020 levels despite economic development.

It is important to understand that negative emissions of ANNEX I countries contain emission reductions from fossil sector decarbonization in ROW countries, which is compensated by financial transfers from ANNEX I to ROW countries. At the same time the ROW emission trajectory reflects what these countries achieve based on their own financial efforts, i.e. emission reductions induced by payments from ANNEX I countries are not considered in this curve. Since the mitigation pathway is globally optimal, this conservative approach is less costly than the alternative compensation of “foreign” fossil emissions by “domestic” negative emissions.

For estimating public income- and expenditure shares (*in* and *ex*, respectively) of specific mitigation pathways, net CO_2_ emissions reflecting the increased mitigation efforts are multiplied by the carbon price and expressed as percentage of the respective share of global GDP (g), which is roughly 60% for ANNEX I countries^3^.

$$g=0.60$$

$$in\left( t \right)= \left\{ \begin{aligned} \frac{\left( P_{CO2}\left( t \right)* {CO2}_{ANNEXI}\left( t \right) \right)}{g*GDP} \\ 0, {CO2}_{ANNEXI}\left( t \right)\leq0 \end{aligned} \right., {CO2}_{ANNEXI}\left( t \right)>0$$

$$ex\left( t \right)= \left\{ \begin{aligned} -\frac{\left( P_{CO2}\left( t \right)* {CO2}_{ANNEXI}\left( t \right) \right)}{g*GDP} \\ 0, {CO2}_{ANNEXI}\left( t \right)\geq0 \end{aligned} \right., {CO2}_{ANNEXI}\left( t \right)<0$$

The expenditure share is consequently a function of 1) the responsibility share (*r*) for contributions to climate change, 2) the current emission share (*ces*) and 3) the share of global GDP (*g*). Obviously a higher historical responsibility results in a higher expenditure share for ANNEX I countries, while a higher share of GDP reduces the expenditure share. Since the current emission share defines the future baseline for ANNEX I countries from which reductions are subtracted, reducing *ces* leads to an increased expenditure share since net ANNEX I emissions need to be lower. Here, one could argue that the share of baseline emissions for ANNEX I countries has to be lower in the future than their current share of emissions (which is the case if allowable emissions per capita are globally the same). Moreover, ANNEX I countries have seen a decrease in the share of global GDP implying that share of global GDP could actually be lower in the future. Both of these aspects suggest that expenditure shares could be larger than our estimates based on static parameters for ANNEX I countries.


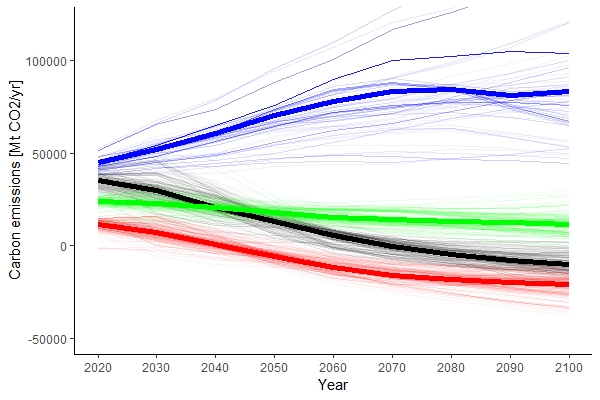


Figure 1

*Global baseline emissions (CO2_base_, blue), emission shares of ANNEX I countries (CO2_ANNEXI_, red) and ROW (CO2_ROW_, green), global net CO_2_ emissions (CO2_scen_, black). ANNEX I countries start from a lower level and go net-negative in around 2040, while ROW emissions stay constant at 2020 levels despite economic growth.*

1. **Non-contribution to climate mitigation**

Equivalent to historical burden sharing, a “freerider narrative” can be constructed. Here, a group of countries representing 75% of global GDP with 85% of current CO_2_ emissions is responsible achieving 100% of the mitigation efforts. The freerider (in this case the United States) with a 15% share of global GDP receives payments to decarbonize from ROW countries. A more realistic set-up would take into account more costly compensation of freerider emissions by negative emissions of other countries, resulting in even higher expenditure shares for the rest of the world than we presented. In Figure 2 global baseline emissions (blue), freerider emissions (green), ROW emissions (red) and global net emissions are illustrated. Again, ROW emissions contain the freerider emission reductions that these countries have to induce through payments to the freerider to achieve the Paris target. Consequently, freerider emissions reflect the efforts done by the freerider’s own means (which is none).


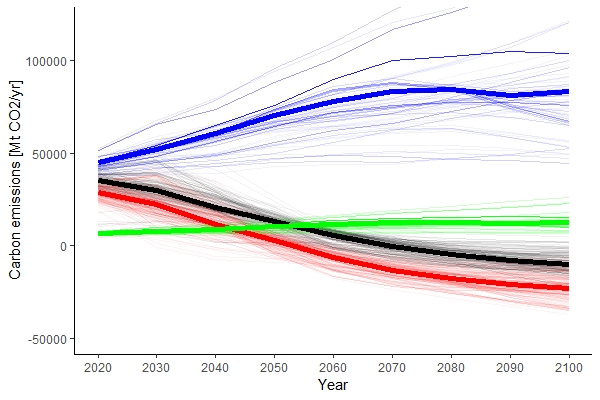


Figure 2

*Global baseline emissions (blue), freerider emissions (green), ROW emissions (red) and global net emissions (black). ROW emissions contain the freerider emission reductions that ROW countries have to induce through payments to the freerider. Freerider emissions reflect only the efforts done by the freerider’s own means, they are therefore equivalent to baseline emissions.*

1. **Calculation of median BECCS negative emissions**

Negative CO_2_ emissions generated by bioenergy with Carbon Capture and Storage (BECCS) are not explicitly reported in the AR5 database. Based on fossil Carbon Capture and Storage (CCS) and BECCS energy production as well as total CCS captured CO_2_ emissions (from bioenergy and fossil sources) we arrive at a rough estimate for the conversion factors (energy to emissions) for BECCS in each scenario by linear regression based on the simplifying assumption that the conversion factors stay constant over time. The conversion factor times BECCS energy production gives the CO_2_ trajectory of BECCS for each scenario. The median scenario as depicted in Figure 3 is in good accordance with the schematic “Managed transition” scenario of the special report on Global Warming of 1.5^o^C recently published by the IPCC.


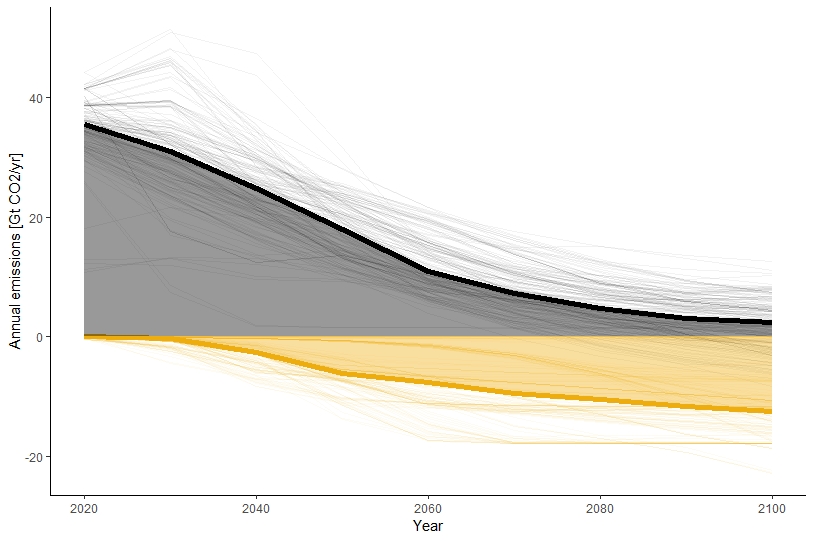


Figure 3

*CO2 captured by BECCS (yellow) and net-CO2 emissions (positive and negative) from all other sectors including agriculture and forestry (black). BECCS negative emissions were derived from BECCS energy consumption and CO2 captured by CCS via linear regression for each scenario.*

1. Mueller, B., Hoehne, N. & Ellermann, C. Differentiating (historic) responsibilities for climate change. *Climate Policy* **9**, 593–611 (2009).

2. Boden, T., Andres, R. & Marland, G. Global, Regional, and National Fossil-Fuel CO2 Emissions (1751 - 2014) (V. 2017). (2017). doi:10.3334/CDIAC/00001_V2017

3. The World Bank. GDP (current US$). Available at: https://data.worldbank.org/indicator/NY.GDP.MKTP.CD. (Accessed: 8th October 2018)
